# 3,5-Diphenyl-1-(quinolin-2-yl)-4,5-dihydro-1*H*-pyrazol-5-ol

**DOI:** 10.1107/S1600536812029340

**Published:** 2012-07-04

**Authors:** Muhd. Hidayat bin Najib, Ai Ling Tan, David J. Young, Seik Weng Ng, Edward R. T. Tiekink

**Affiliations:** a Faculty of Science, Universiti Brunei Darussalam, Jalan Tungku Link BE 1410, Negara Brunei Darussalam; b Department of Chemistry, University of Malaya, 50603 Kuala Lumpur, Malaysia; c Chemistry Department and Faculty of Science, King Abdulaziz University, PO Box 80203 Jeddah, Saudi Arabia

## Abstract

In the title compound, C_24_H_19_N_3_O, the pyrazole ring is close to being planar (r.m.s. deviation of the five fitted atoms = 0.062 Å), and each of the *N*-bound quinoline ring [dihedral angle = 9.90 (7)°] and the *C*-bound phenyl ring in the 3-position is close to being coplanar [dihedral angle = 8.87 (9)°]. However, the phenyl ring in the 5-position forms a dihedral angle of 72.31 (9)°. The hy­droxy group forms an intra­molecular hydrogen bond to the quinoline N atom. In the crystal, mol­ecules are connected into supra­molecular layers two mol­ecules thick in the *bc* plane by C—H⋯O and C—H⋯π inter­actions.

## Related literature

For applications of coordination complexes of hydrazones as organic light emitting diodes and supra­molecular magnetic clusters, see: Zhang *et al.* (2011 ▶, 2012 ▶). For the synthesis of hydrazones, see: Gupta *et al.* (2007 ▶). For background to and the synthesis of the target mol­ecules, see: Najib *et al.* (2012*a* ▶,*b* ▶,*c* ▶)
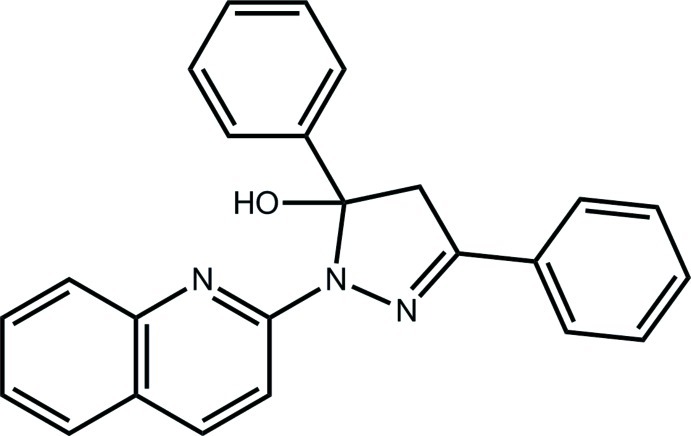


## Experimental

### 

#### Crystal data

C_24_H_19_N_3_O
*M* *_r_* = 365.42
Monoclinic, 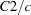

*a* = 30.505 (2) Å
*b* = 7.8881 (4) Å
*c* = 16.5191 (12) Å
β = 113.718 (9)°
*V* = 3639.1 (4) Å^3^
*Z* = 8
Mo *K*α radiation
μ = 0.08 mm^−1^
*T* = 100 K
0.35 × 0.30 × 0.25 mm

#### Data collection

Agilent SuperNova Dual diffractometer with an Atlas detector
Absorption correction: multi-scan (*CrysAlis PRO*; Agilent, 2012 ▶) *T*_min_ = 0.784, *T*_max_ = 1.000
12177 measured reflections
4209 independent reflections
3419 reflections with *I* > 2σ(*I*)
*R*_int_ = 0.033

#### Refinement

*R*[*F* ^2^ > 2σ(*F* ^2^)] = 0.048
*wR*(*F* ^2^) = 0.130
*S* = 1.07
4209 reflections
257 parameters
1 restraint
H atoms treated by a mixture of independent and constrained refinement
Δρ_max_ = 0.30 e Å^−3^
Δρ_min_ = −0.32 e Å^−3^

### 

Data collection: *CrysAlis PRO* (Agilent, 2012 ▶); cell refinement: *CrysAlis PRO*; data reduction: *CrysAlis PRO*; program(s) used to solve structure: *SHELXS97* (Sheldrick, 2008 ▶); program(s) used to refine structure: *SHELXL97* (Sheldrick, 2008 ▶); molecular graphics: *ORTEP-3 for Windows* (Farrugia, 1997 ▶) and *DIAMOND* (Brandenburg, 2006 ▶); software used to prepare material for publication: *publCIF* (Westrip, 2010 ▶).

## Supplementary Material

Crystal structure: contains datablock(s) global, I. DOI: 10.1107/S1600536812029340/sj5251sup1.cif

Structure factors: contains datablock(s) I. DOI: 10.1107/S1600536812029340/sj5251Isup2.hkl

Supplementary material file. DOI: 10.1107/S1600536812029340/sj5251Isup3.cml

Additional supplementary materials:  crystallographic information; 3D view; checkCIF report

## Figures and Tables

**Table 1:** Hydrogen-bond geometry (Å, °) *Cg*1 and *Cg*2 are the centroids of the C1–C6 and N1,C1,C6–C9 rings, respectively.

*D*—H⋯*A*	*D*—H	H⋯*A*	*D*⋯*A*	*D*—H⋯*A*
O1—H1*o*⋯N1	0.87 (1)	2.15 (2)	2.8149 (19)	133 (3)
C3—H3⋯O1^i^	0.95	2.50	3.298 (2)	142
C11—H11*A*⋯*Cg*1^ii^	0.99	2.92	3.8528 (18)	157
C17—H17⋯*Cg*2^iii^	0.95	2.58	3.4426 (18)	151
C24—H24⋯*Cg*2^ii^	0.95	2.97	3.6239 (18)	127

Symmetry codes: (i) 
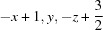
; (ii) 
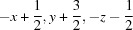
; (iii) 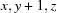.

**Fig. 1.**
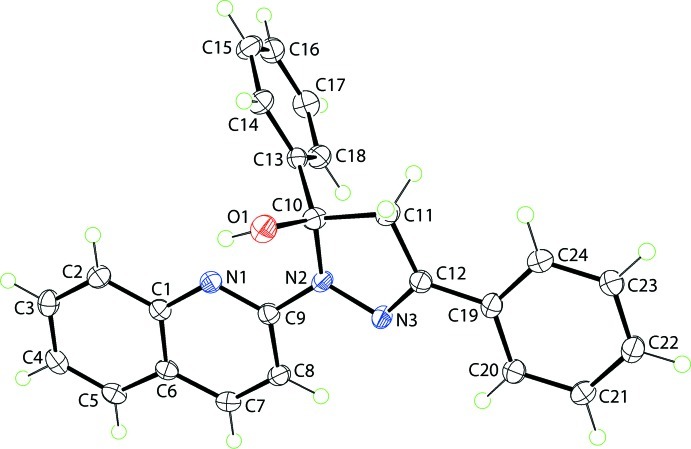
The molecular structure of (I) showing the atom-labelling scheme and displacement ellipsoids at the 50% probability level.

**Fig. 2.**
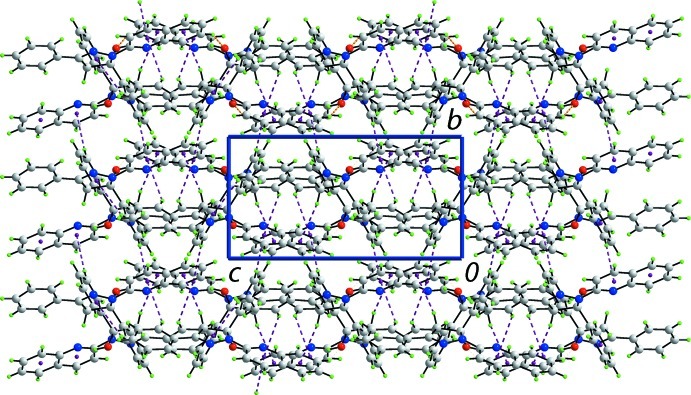
A view of the supramolecular array in the bc plane in (I). The C—H···O and C—H···π interactions are shown as orange and purple dashed lines, respectively.

**Fig. 3.**
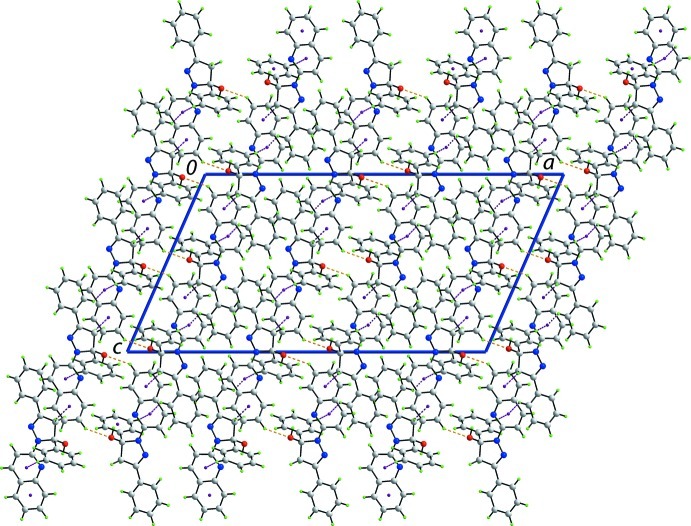
A view of the unit-cell contents of (I) in projection down the b axis. The C—H···O and C—H···π interactions are shown as orange and purple dashed lines, respectively.

## supplementary crystallographic information

### Comment

We have previously prepared 3,5-dimethyl-1-(2'-quinolyl)-pyrazole (Najib *et al.*, 2012*c*) and a related *Cinchonine* derived ligand (Zhang *et al.*, 2011) for the synthesis of photoluminescent zinc (Najib *et al.*, 2012*a*; Najib *et al.*, 2012*b*) and iridium complexes (Zhang *et al.*, 2012). These ligands are made by the condensation of the corresponding hydrazine (Najib *et al.*, 2012*a*) with a β-diketone (Gupta *et al.*, 2007). In our attempted synthesis of 3,5-diphenyl-1-(2'-quinolyl)-pyrazole using the same procedure, we were surprised to prepare the title compound, (I), presumably from initial condensation but only partial dehydration. The reluctance of this benzylic, tertiary alcohol to undergo dehydration may be due to the resulting unfavourable proximity of the phenyl and quinoline groups.

In (I), the pyrazolyl ring has an envelope configuration with the C10 atom being the flap atom. However, the distortion from planarity is relatively minor with the r.m.s. deviation = 0.062 Å and maximum deviations of 0.051 (1) Å for the N1 atom and -0.052 (2) Å for the C10 atom. The N2-bound quinolinyl ring (r.m.s. deviation = 0.009 Å) forms a dihedral angle of 9.90 (7)° with the pyrazolyl plane. The C12-bound phenyl ring is almost co-planar with the pyrazolyl plane [dihedral angle = 8.87 (9)°) whereas the C10-bound phenyl ring forms a dihedral angle of 72.31 (9)°. The latter projects to one side of the pyrazolyl plane and the hydroxy group to the other. The hydroxy group forms an intramolecular hydrogen bond to the quinolinyl-N1 atom, Table 1.

In the crystal packing, C—H···O interactions link molecules into centrosymmetric dimers *via* 18-membered {···HC_3_NCNCO}_2_ synthons, Table 1. These are connected into supramolecular layers two molecules thick in the *bc* plane *via* C—H···π interactions, Fig. 2 and Table 1. Layers stack along the *a* axis without specific interactions between them.

### Experimental

Ethanol (25 ml) was added to a mixture of 2-hydrazinylquinoline (0.08 g) and dibenzoylmethane (0.23 g) and the resulting solution was refluxed for 48 h. The solvent was removed to obtain an orange residue that was recrystallized from toluene to yield 0.063 g of orange crystals. A second recrystallization from toluene produced 0.035 g (19.1%) of orange crystals. Melting point: 503 K. IR ν/cm^-1^: 3409, 3061, 3028, 1616, 1602, 1559, 1507, 1480, 1447, 1432, 1405, 1371, 1343, 1326, 1302, 1263, 1248, 1233, 1170, 1149, 1070, 1050, 856, 828, 756, 698, 692. ^1^H NMR 400 MHz (CDCl_3_) δ: 8.00 (1*H*, d), 7.80 (3*H*, m), 7.58 (3*H*, m), 7.42 (5*H*, m), 7.30 (1*H*, m), 7.21 (2*H*, m), 7.15(1*H*, m), 3.83 (1*H*, d), 3.50 (1*H*, d), 1.25 (1*H*, s).

### Refinement

Carbon-bound H-atoms were placed in calculated positions [C—H = 0.95–0.99 Å, *U*_iso_(H) = 1.2*U*_eq_(C)] and were included in the refinement in the riding model approximation. The oxygen-bound H-atom was refined with O—H = 0.84±0.01 Å and free *U*_iso_.

### Crystal data

**Table:**

C_24_H_19_N_3_O	*F*(000) = 1536
*M**_r_* = 365.42	*D*_x_ = 1.334 Mg m^−^^3^
Monoclinic, *C*2/*c*	Mo *K*α radiation, λ = 0.71073 Å
Hall symbol: -C 2yc	Cell parameters from 4159 reflections
*a* = 30.505 (2) Å	θ = 2.4–27.5°
*b* = 7.8881 (4) Å	µ = 0.08 mm^−^^1^
*c* = 16.5191 (12) Å	*T* = 100 K
β = 113.718 (9)°	Block, yellow
*V* = 3639.1 (4) Å^3^	0.35 × 0.30 × 0.25 mm
*Z* = 8	

### Data collection

**Table:**

Agilent SuperNova Dual diffractometer with an Atlas detector	4209 independent reflections
Radiation source: fine-focus sealed tube	3419 reflections with *I* > 2σ(*I*)
Mirror monochromator	*R*_int_ = 0.033
Detector resolution: 10.4041 pixels mm^-1^	θ_max_ = 27.6°, θ_min_ = 2.5°
ω scan	*h* = −30→39
Absorption correction: multi-scan (*CrysAlis PRO*; Agilent, 2012)	*k* = −9→10
*T*_min_ = 0.784, *T*_max_ = 1.000	*l* = −21→14
12177 measured reflections	

### Refinement

**Table:**

Refinement on *F*^2^	Primary atom site location: structure-invariant direct methods
Least-squares matrix: full	Secondary atom site location: difference Fourier map
*R*[*F*^2^ > 2σ(*F*^2^)] = 0.048	Hydrogen site location: inferred from neighbouring sites
*wR*(*F*^2^) = 0.130	H atoms treated by a mixture of independent and constrained refinement
*S* = 1.07	*w* = 1/[σ^2^(*F*_o_^2^) + (0.0534*P*)^2^ + 3.4025*P*] where *P* = (*F*_o_^2^ + 2*F*_c_^2^)/3
4209 reflections	(Δ/σ)_max_ < 0.001
257 parameters	Δρ_max_ = 0.30 e Å^−^^3^
1 restraint	Δρ_min_ = −0.32 e Å^−^^3^

### Special details

**Table:**

Geometry. All e.s.d.'s (except the e.s.d. in the dihedral angle between two l.s. planes) are estimated using the full covariance matrix. The cell e.s.d.'s are taken into account individually in the estimation of e.s.d.'s in distances, angles and torsion angles; correlations between e.s.d.'s in cell parameters are only used when they are defined by crystal symmetry. An approximate (isotropic) treatment of cell e.s.d.'s is used for estimating e.s.d.'s involving l.s. planes.
Refinement. Refinement of *F*^2^ against ALL reflections. The weighted *R*-factor *wR* and goodness of fit *S* are based on *F*^2^, conventional *R*-factors *R* are based on *F*, with *F* set to zero for negative *F*^2^. The threshold expression of *F*^2^ > σ(*F*^2^) is used only for calculating *R*-factors(gt) *etc*. and is not relevant to the choice of reflections for refinement. *R*-factors based on *F*^2^ are statistically about twice as large as those based on *F*, and *R*- factors based on ALL data will be even larger.

### Fractional atomic coordinates and isotropic or equivalent isotropic displacement parameters (Å^2^)

**Table:**

	*x*	*y*	*z*	*U*_iso_*/*U*_eq_	
O1	0.43944 (4)	0.27304 (15)	0.51758 (8)	0.0265 (3)	
H1o	0.4449 (11)	0.250 (4)	0.5723 (9)	0.087 (11)*	
N1	0.40135 (5)	0.26810 (16)	0.64704 (9)	0.0201 (3)	
N2	0.36017 (5)	0.33420 (17)	0.50098 (9)	0.0208 (3)	
N3	0.32336 (5)	0.29868 (16)	0.42006 (9)	0.0210 (3)	
C1	0.40233 (6)	0.20442 (18)	0.72467 (10)	0.0187 (3)	
C2	0.44529 (6)	0.2169 (2)	0.80119 (11)	0.0225 (3)	
H2	0.4724	0.2708	0.7980	0.027*	
C3	0.44810 (6)	0.1519 (2)	0.88010 (11)	0.0241 (4)	
H3	0.4772	0.1607	0.9311	0.029*	
C4	0.40834 (6)	0.0723 (2)	0.88630 (11)	0.0246 (4)	
H4	0.4107	0.0275	0.9413	0.030*	
C5	0.36626 (6)	0.05912 (19)	0.81328 (11)	0.0232 (3)	
H5	0.3395	0.0054	0.8179	0.028*	
C6	0.36217 (6)	0.12429 (18)	0.73125 (10)	0.0195 (3)	
C7	0.31958 (6)	0.1126 (2)	0.65227 (11)	0.0218 (3)	
H7	0.2921	0.0580	0.6534	0.026*	
C8	0.31823 (6)	0.17893 (19)	0.57593 (11)	0.0213 (3)	
H8	0.2899	0.1730	0.5232	0.026*	
C9	0.36047 (6)	0.25830 (19)	0.57660 (10)	0.0193 (3)	
C10	0.40389 (6)	0.3997 (2)	0.49355 (11)	0.0215 (3)	
C11	0.38509 (6)	0.4283 (2)	0.39280 (10)	0.0234 (4)	
H11A	0.3836	0.5507	0.3786	0.028*	
H11B	0.4056	0.3703	0.3677	0.028*	
C12	0.33582 (5)	0.35170 (19)	0.35852 (10)	0.0196 (3)	
C13	0.42182 (5)	0.56032 (19)	0.54739 (10)	0.0185 (3)	
C14	0.47014 (6)	0.6033 (2)	0.57873 (11)	0.0228 (3)	
H14	0.4921	0.5291	0.5690	0.027*	
C15	0.48632 (6)	0.7545 (2)	0.62411 (12)	0.0275 (4)	
H15	0.5192	0.7839	0.6449	0.033*	
C16	0.45464 (6)	0.8621 (2)	0.63909 (11)	0.0273 (4)	
H16	0.4658	0.9649	0.6706	0.033*	
C17	0.40668 (6)	0.8200 (2)	0.60811 (12)	0.0283 (4)	
H17	0.3848	0.8937	0.6184	0.034*	
C18	0.39055 (6)	0.6704 (2)	0.56208 (11)	0.0245 (4)	
H18	0.3575	0.6429	0.5402	0.029*	
C19	0.30291 (5)	0.33954 (19)	0.26559 (10)	0.0194 (3)	
C20	0.25909 (6)	0.2537 (2)	0.24096 (11)	0.0213 (3)	
H20	0.2515	0.1979	0.2846	0.026*	
C21	0.22711 (6)	0.2503 (2)	0.15352 (11)	0.0233 (3)	
H21	0.1975	0.1919	0.1372	0.028*	
C22	0.23785 (6)	0.3313 (2)	0.08928 (11)	0.0240 (3)	
H22	0.2154	0.3305	0.0294	0.029*	
C23	0.28120 (6)	0.4133 (2)	0.11249 (11)	0.0238 (4)	
H23	0.2887	0.4674	0.0682	0.029*	
C24	0.31400 (6)	0.4175 (2)	0.20006 (11)	0.0223 (3)	
H24	0.3439	0.4731	0.2154	0.027*	

### Atomic displacement parameters (Å^2^)

**Table:**

	*U*^11^	*U*^22^	*U*^33^	*U*^12^	*U*^13^	*U*^23^
O1	0.0252 (6)	0.0256 (6)	0.0271 (7)	0.0021 (5)	0.0087 (5)	−0.0023 (5)
N1	0.0211 (7)	0.0193 (6)	0.0190 (7)	−0.0010 (5)	0.0071 (5)	−0.0013 (5)
N2	0.0180 (6)	0.0245 (7)	0.0171 (6)	−0.0051 (5)	0.0042 (5)	−0.0010 (5)
N3	0.0198 (7)	0.0213 (7)	0.0175 (7)	−0.0006 (5)	0.0031 (5)	−0.0006 (5)
C1	0.0216 (8)	0.0162 (7)	0.0194 (8)	0.0011 (6)	0.0094 (6)	−0.0018 (6)
C2	0.0209 (8)	0.0239 (8)	0.0231 (8)	−0.0005 (6)	0.0094 (6)	−0.0018 (6)
C3	0.0253 (8)	0.0263 (8)	0.0188 (8)	0.0043 (6)	0.0068 (6)	−0.0015 (6)
C4	0.0344 (9)	0.0197 (8)	0.0218 (8)	0.0042 (6)	0.0134 (7)	0.0031 (6)
C5	0.0286 (8)	0.0165 (7)	0.0272 (8)	−0.0003 (6)	0.0140 (7)	0.0001 (6)
C6	0.0229 (8)	0.0140 (7)	0.0232 (8)	0.0012 (6)	0.0108 (6)	−0.0015 (6)
C7	0.0221 (8)	0.0187 (7)	0.0273 (8)	−0.0026 (6)	0.0128 (7)	−0.0016 (6)
C8	0.0189 (7)	0.0198 (7)	0.0233 (8)	−0.0015 (6)	0.0066 (6)	−0.0020 (6)
C9	0.0214 (8)	0.0170 (7)	0.0194 (8)	0.0003 (6)	0.0083 (6)	−0.0009 (6)
C10	0.0210 (8)	0.0222 (8)	0.0217 (8)	−0.0031 (6)	0.0088 (6)	−0.0024 (6)
C11	0.0227 (8)	0.0258 (8)	0.0197 (8)	−0.0047 (6)	0.0065 (6)	−0.0027 (6)
C12	0.0205 (8)	0.0173 (7)	0.0202 (8)	0.0012 (6)	0.0074 (6)	−0.0004 (6)
C13	0.0210 (7)	0.0196 (7)	0.0146 (7)	−0.0023 (6)	0.0067 (6)	0.0003 (6)
C14	0.0199 (8)	0.0245 (8)	0.0239 (8)	−0.0003 (6)	0.0085 (6)	−0.0036 (6)
C15	0.0212 (8)	0.0278 (9)	0.0292 (9)	−0.0051 (7)	0.0057 (7)	−0.0041 (7)
C16	0.0335 (9)	0.0216 (8)	0.0233 (8)	−0.0019 (7)	0.0077 (7)	−0.0040 (6)
C17	0.0310 (9)	0.0256 (9)	0.0308 (9)	0.0052 (7)	0.0150 (7)	−0.0023 (7)
C18	0.0200 (8)	0.0264 (8)	0.0273 (9)	0.0005 (6)	0.0096 (7)	0.0010 (7)
C19	0.0193 (7)	0.0180 (7)	0.0186 (7)	0.0039 (6)	0.0051 (6)	−0.0017 (6)
C20	0.0214 (8)	0.0220 (8)	0.0202 (8)	0.0001 (6)	0.0081 (6)	−0.0006 (6)
C21	0.0182 (8)	0.0256 (8)	0.0245 (8)	−0.0021 (6)	0.0069 (6)	−0.0015 (6)
C22	0.0229 (8)	0.0256 (8)	0.0198 (8)	0.0010 (6)	0.0048 (6)	−0.0010 (6)
C23	0.0260 (8)	0.0249 (8)	0.0207 (8)	−0.0015 (6)	0.0097 (7)	0.0012 (6)
C24	0.0201 (8)	0.0218 (8)	0.0246 (8)	−0.0019 (6)	0.0086 (6)	−0.0020 (6)

### Geometric parameters (Å, º)

**Table:**

O1—C10	1.409 (2)	C11—H11A	0.9900
O1—H1o	0.871 (10)	C11—H11B	0.9900
N1—C9	1.3220 (19)	C12—C19	1.461 (2)
N1—C1	1.366 (2)	C13—C18	1.382 (2)
N2—C9	1.382 (2)	C13—C14	1.393 (2)
N2—N3	1.3850 (17)	C14—C15	1.389 (2)
N2—C10	1.481 (2)	C14—H14	0.9500
N3—C12	1.290 (2)	C15—C16	1.381 (2)
C1—C2	1.411 (2)	C15—H15	0.9500
C1—C6	1.421 (2)	C16—C17	1.382 (2)
C2—C3	1.371 (2)	C16—H16	0.9500
C2—H2	0.9500	C17—C18	1.383 (2)
C3—C4	1.405 (2)	C17—H17	0.9500
C3—H3	0.9500	C18—H18	0.9500
C4—C5	1.367 (2)	C19—C24	1.400 (2)
C4—H4	0.9500	C19—C20	1.405 (2)
C5—C6	1.407 (2)	C20—C21	1.380 (2)
C5—H5	0.9500	C20—H20	0.9500
C6—C7	1.426 (2)	C21—C22	1.386 (2)
C7—C8	1.350 (2)	C21—H21	0.9500
C7—H7	0.9500	C22—C23	1.381 (2)
C8—C9	1.429 (2)	C22—H22	0.9500
C8—H8	0.9500	C23—C24	1.389 (2)
C10—C13	1.518 (2)	C23—H23	0.9500
C10—C11	1.543 (2)	C24—H24	0.9500
C11—C12	1.503 (2)		
			
C10—O1—H1o	104 (2)	C12—C11—H11B	111.1
C9—N1—C1	117.48 (13)	C10—C11—H11B	111.1
C9—N2—N3	119.53 (12)	H11A—C11—H11B	109.1
C9—N2—C10	123.18 (12)	N3—C12—C19	120.74 (14)
N3—N2—C10	113.46 (12)	N3—C12—C11	113.60 (14)
C12—N3—N2	108.27 (13)	C19—C12—C11	125.63 (14)
N1—C1—C2	118.47 (14)	C18—C13—C14	118.91 (14)
N1—C1—C6	122.77 (14)	C18—C13—C10	121.07 (14)
C2—C1—C6	118.76 (14)	C14—C13—C10	119.93 (14)
C3—C2—C1	120.37 (15)	C15—C14—C13	120.18 (15)
C3—C2—H2	119.8	C15—C14—H14	119.9
C1—C2—H2	119.8	C13—C14—H14	119.9
C2—C3—C4	120.71 (15)	C16—C15—C14	120.13 (16)
C2—C3—H3	119.6	C16—C15—H15	119.9
C4—C3—H3	119.6	C14—C15—H15	119.9
C5—C4—C3	120.08 (15)	C15—C16—C17	119.90 (16)
C5—C4—H4	120.0	C15—C16—H16	120.1
C3—C4—H4	120.0	C17—C16—H16	120.1
C4—C5—C6	120.68 (16)	C16—C17—C18	119.89 (16)
C4—C5—H5	119.7	C16—C17—H17	120.1
C6—C5—H5	119.7	C18—C17—H17	120.1
C5—C6—C1	119.40 (14)	C13—C18—C17	120.99 (15)
C5—C6—C7	123.54 (15)	C13—C18—H18	119.5
C1—C6—C7	117.06 (14)	C17—C18—H18	119.5
C8—C7—C6	120.24 (15)	C24—C19—C20	119.05 (14)
C8—C7—H7	119.9	C24—C19—C12	120.41 (14)
C6—C7—H7	119.9	C20—C19—C12	120.51 (15)
C7—C8—C9	118.28 (14)	C21—C20—C19	120.14 (15)
C7—C8—H8	120.9	C21—C20—H20	119.9
C9—C8—H8	120.9	C19—C20—H20	119.9
N1—C9—N2	115.56 (14)	C20—C21—C22	120.45 (15)
N1—C9—C8	124.12 (14)	C20—C21—H21	119.8
N2—C9—C8	120.31 (14)	C22—C21—H21	119.8
O1—C10—N2	110.25 (13)	C23—C22—C21	119.90 (15)
O1—C10—C13	111.82 (12)	C23—C22—H22	120.1
N2—C10—C13	111.52 (13)	C21—C22—H22	120.1
O1—C10—C11	108.52 (13)	C22—C23—C24	120.55 (16)
N2—C10—C11	100.60 (12)	C22—C23—H23	119.7
C13—C10—C11	113.56 (13)	C24—C23—H23	119.7
C12—C11—C10	103.23 (13)	C23—C24—C19	119.88 (15)
C12—C11—H11A	111.1	C23—C24—H24	120.1
C10—C11—H11A	111.1	C19—C24—H24	120.1
			
C9—N2—N3—C12	−165.92 (14)	N2—C10—C11—C12	−7.55 (15)
C10—N2—N3—C12	−7.26 (17)	C13—C10—C11—C12	−126.82 (14)
C9—N1—C1—C2	−178.59 (14)	N2—N3—C12—C19	−176.76 (13)
C9—N1—C1—C6	2.3 (2)	N2—N3—C12—C11	1.48 (18)
N1—C1—C2—C3	−178.78 (14)	C10—C11—C12—N3	4.29 (18)
C6—C1—C2—C3	0.4 (2)	C10—C11—C12—C19	−177.58 (14)
C1—C2—C3—C4	−0.2 (2)	O1—C10—C13—C18	−153.52 (15)
C2—C3—C4—C5	−0.1 (2)	N2—C10—C13—C18	−29.6 (2)
C3—C4—C5—C6	0.2 (2)	C11—C10—C13—C18	83.26 (19)
C4—C5—C6—C1	0.0 (2)	O1—C10—C13—C14	30.0 (2)
C4—C5—C6—C7	179.05 (15)	N2—C10—C13—C14	153.99 (14)
N1—C1—C6—C5	178.84 (14)	C11—C10—C13—C14	−93.20 (18)
C2—C1—C6—C5	−0.3 (2)	C18—C13—C14—C15	0.1 (2)
N1—C1—C6—C7	−0.3 (2)	C10—C13—C14—C15	176.68 (15)
C2—C1—C6—C7	−179.38 (14)	C13—C14—C15—C16	0.6 (3)
C5—C6—C7—C8	179.68 (15)	C14—C15—C16—C17	−0.6 (3)
C1—C6—C7—C8	−1.3 (2)	C15—C16—C17—C18	−0.1 (3)
C6—C7—C8—C9	0.7 (2)	C14—C13—C18—C17	−0.9 (2)
C1—N1—C9—N2	175.86 (13)	C10—C13—C18—C17	−177.38 (15)
C1—N1—C9—C8	−2.9 (2)	C16—C17—C18—C13	0.9 (3)
N3—N2—C9—N1	165.54 (13)	N3—C12—C19—C24	171.36 (14)
C10—N2—C9—N1	9.0 (2)	C11—C12—C19—C24	−6.6 (2)
N3—N2—C9—C8	−15.6 (2)	N3—C12—C19—C20	−6.8 (2)
C10—N2—C9—C8	−172.13 (14)	C11—C12—C19—C20	175.19 (15)
C7—C8—C9—N1	1.5 (2)	C24—C19—C20—C21	−1.5 (2)
C7—C8—C9—N2	−177.26 (14)	C12—C19—C20—C21	176.65 (15)
C9—N2—C10—O1	52.69 (19)	C19—C20—C21—C22	0.0 (2)
N3—N2—C10—O1	−105.08 (14)	C20—C21—C22—C23	1.3 (3)
C9—N2—C10—C13	−72.15 (18)	C21—C22—C23—C24	−0.9 (3)
N3—N2—C10—C13	130.07 (13)	C22—C23—C24—C19	−0.7 (2)
C9—N2—C10—C11	167.12 (14)	C20—C19—C24—C23	1.9 (2)
N3—N2—C10—C11	9.34 (16)	C12—C19—C24—C23	−176.29 (14)
O1—C10—C11—C12	108.17 (14)		

### Hydrogen-bond geometry (Å, º)

Cg1 and Cg2 are the centroids of the C1–C6 and N1,C1,C6–C9 rings, respectively.

**Table:**

*D*—H···*A*	*D*—H	H···*A*	*D*···*A*	*D*—H···*A*
O1—H1*o*···N1	0.87 (1)	2.15 (2)	2.8149 (19)	133 (3)
C3—H3···O1^i^	0.95	2.50	3.298 (2)	142
C11—H11*A*···*Cg*1^ii^	0.99	2.92	3.8528 (18)	157
C17—H17···*Cg*2^iii^	0.95	2.58	3.4426 (18)	151
C24—H24···*Cg*2^ii^	0.95	2.97	3.6239 (18)	127

Symmetry codes: (i) −*x*+1, *y*, −*z*+3/2; (ii) −*x*+1/2, *y*+3/2, −*z*−1/2; (iii) *x*, *y*+1, *z*.

## References

[bb1] Agilent (2012). *CrysAlis PRO* Agilent Technologies, Yarnton, England.

[bb2] Brandenburg, K. (2006). *DIAMOND* Crystal Impact GbR, Bonn, Germany.

[bb3] Farrugia, L. J. (1997). *J. Appl. Cryst.* **30**, 565.

[bb4] Gupta, L. K., Bansal, U. & Chandra, S. (2007). *Spectrochim. Acta Part A*, **66**, 972–975. 10.1016/j.saa.2006.04.035 16872867

[bb5] Najib, M. H. bin, Tan, A. L., Young, D. J., Ng, S. W. & Tiekink, E. R. T. (2012*a*). *Acta Cryst.* E**68**, m571–m572. 10.1107/S1600536812014390 PMC3344321 22590087

[bb6] Najib, M. H. bin, Tan, A. L., Young, D. J., Ng, S. W. & Tiekink, E. R. T. (2012*b*). *Acta Cryst.* E**68**, m897–m898. 10.1107/S1600536812025664 PMC3393171 22807739

[bb7] Najib, M. H. bin, Tan, A. L., Young, D. J., Ng, S. W. & Tiekink, E. R. T. (2012*c*). *Acta Cryst.* E**68**, o2138. 10.1107/S1600536812026906 PMC3393948 22798813

[bb8] Sheldrick, G. M. (2008). *Acta Cryst.* A**64**, 112–122. 10.1107/S0108767307043930 18156677

[bb9] Westrip, S. P. (2010). *J. Appl. Cryst.* **43**, 920–925.

[bb10] Zhang, W. H., Hu, J. J., Chi, Y., Young, D. J. & Hor, T. S. A. (2011). *Organometallics*, **30**, 2137–2143.

[bb11] Zhang, W. H., Zhang, X. H., Tan, A. L., Yong, M. A., Young, D. J. & Hor, T. S. A. (2012). *Organometallics*, **31**, 553–559.

